# Impact of DPP-4 inhibitors on plasma levels of BNP and NT-pro-BNP in type 2 diabetes mellitus

**DOI:** 10.1186/s13098-022-00797-x

**Published:** 2022-02-14

**Authors:** Liying Mu, Zhuo Wang, Jinmei Ren, Xiaowei Xiong, Zening Jin, Xin Liu

**Affiliations:** 1 Department of Cardiology, Beijing Tiantan Hospital, Capital Medical University, Beijing, China; 2 Department of Gastroenterology, Beijing Tiantan Hospital, Capital Medical University, Beijing, China; 3 Department of Pharmacy, Qingpu Branch of Zhongshan, Fudan University, Shanghai, China; 4 Department of Pharmacy, Beijing Tiantan Hospital, Capital Medical University, Beijing, China; 5 Department of Cardiology and Macrovascular Disease, Beijing Tiantan Hospital, Capital Medical University, Beijing, China

**Keywords:** Dipeptidyl peptidase-4 inhibitors, BNP, NT-pro-BNP, Type 2 diabetes mellitus, Randomized controlled trials

## Abstract

**Background:**

Dipeptidyl peptidase-4 inhibitors (DPP-4i) decrease glucose levels by regulating incretin peptides in type 2 diabetes mellitus (T2DM). This study aimed to determine the modulatory effect of DPP-4i on brain natriuretic peptide (BNP) and N-terminal pro-brain natriuretic peptide (NT-pro-BNP) in patients with T2DM.

**Methods:**

PubMed, Embase and the Cochrane Library were systematically searched to identify randomized controlled trials (RCTs) evaluating the impact of DPP-4i on BNP or NT-pro-BNP. A fixed- or random-effects model was used for quantitative analysis, according to the heterogeneity. Sensitivity analysis and publication bias were performed using standard methods.

**Results:**

Nine trials with 3056 patients with T2DM were included. Meta-analysis revealed that DPP-4i did not significantly modulate the BNP (0.21 pg/mL, 95% CI − 2.36–2.79) or NT-pro-BNP level (− 7.34 pg/mL, 95% CI − 24.27–9.59). DPP-4i demonstrated no stronger effect on modulating BNP (5.17 pg/mL, 95% CI − 7.48–17.82) or NT-pro-BNP (− 9.95 pg/mL, 95% CI − 44.61–24.71) than active comparators. Pooled analysis was robust and stable after sensitivity analysis.

**Conclusions:**

DPP-4i exhibits no significant effect on modulating BNP or NT-pro-BNP and shows no stronger effect than traditional antidiabetic agents in T2DM.

**Supplementary Information:**

The online version contains supplementary material available at 10.1186/s13098-022-00797-x.

## Introduction

Dipeptidyl peptidase-4 inhibitors (DPP-4is) have been developed as oral antidiabetic agents in type 2 diabetes mellitus (T2DM), as they prevent the degradation of incretins such as glucagon-like peptide 1 (GLP-1) and glucose-dependent insulinotropic polypeptide (GIP) [1]. DPP-4is have a favorable effect on lowering glucose levels without increasing the risk of hypoglycemia or weight gain [2]. However, in the past 2 decades, cardiovascular safety issues have been taken into consideration for antidiabetic agents since reports about the increased risk of cardiovascular outcomes with rosiglitazone [3].

In fact, the Food and Drug Administration (FDA) stated that new agents for treating T2DM must have been tested for cardiovascular safety in clinical trials [4]. Therefore, DPP-4is have been studied in a serious-cardiovascular-outcome trial. According to its results, different DPP-4is had different effects on cardiovascular events. Like linagliptin, sitagliptin had neutral cardiovascular aspects, and alogliptin in the EXAMINE trial did not demonstrate a beneficial effect [5]. Saxagliptin in the SAVOR-TIMI 53 trial even showed a higher risk of hospitalization for heart failure [6]. Therefore, clinicians face confusing information as to how to select a suitable DPP-4i for treating T2DM patients with high cardiovascular risk.

There are substrates of DPP-4 beyond glucagon-like peptides that have been proven to have cardiovascular effects, such as brain natriuretic peptide (BNP) [7]. BNP and N-terminal pro-brain natriuretic peptide (NT-pro-BNP) are released as products of cardiomyocytes in cardiovascular disease. They have been recognized as valuable biomarkers for heart failure in clinical practice [8]. Since no association between BNP or NT-pro-BNP and DPP-4is in T2DM has been established, the current meta-analysis was performed to identify the impact of DPP-4is on BNP and NT-pro-BNP in type 2 diabetes.

## Methods

### Literature search strategy

This meta-analysis was conducted according to the Preferred Reporting Items for Systematic Reviews and Meta-Analysis (PRISMA) guidelines [9]. We searched PubMed, Embase and the Cochrane Library for randomized controlled trials (RCTs) with the key words “sitagliptin” OR “vildagliptin” OR “teneligliptin” OR “saxagliptin” OR “linagliptin” OR “alogliptin” from inception to April 30, 2021. The search was limited to human studies, and no language restriction was applied. Two authors independently performed the search by applying the inclusion and exclusion criteria and extracted the data from the retrieved studies.

### Study selection

Two authors independently reviewed studies based on the titles, abstracts, and keywords to ensure that no relevant study was missed. Eligible studies met all the following criteria: (1) randomized controlled studies comparing DPP-4is with placebo or other diabetic medication; (2) studies with information on BNP or NT-pro-BNP levels at baseline and at the end of follow-up in each group or providing the net change values; and (3) studies conducted in patients with T2DM. The exclusion criteria were as follows: (1) studies conducted in healthy volunteers; (2) studies not in humans; (3) narrative reviews or abstracts; and (4) studies that did not provide data on the levels of BNP or NT-pro-BNP at baseline or at the end of follow-up. The inclusion and exclusion criteria were evaluated objectively by two reviewers. The reference lists of eligible articles were hand-searched, and corresponding authors were contacted if relevant information was missing.

### Data extraction

Two reviewers extracted the data and entered them into a designed form. Detailed information was listed, including first author, publication year, country origin, sample size, ratio of men to women, body mass index, mean age, diabetes duration, medication intervention, therapy duration and serum BNP/NT-pro-BNP concentrations at baseline. In studies with different treatment durations, the longest therapy duration was extracted for statistical analysis. The primary outcome measure was a net change in the BNP/NT-pro-BNP concentrations.

### Quality evaluation

Study quality was evaluated according to the Cochrane Reviewers’ Handbook. The items used for the evaluation of each study included random sequence generation, allocation concealment, blinding of participants and personnel, blinding of outcome assessment, incomplete outcome data, selective outcome reporting, and other potential sources of bias. According to the Cochrane Handbook, a judgment of ‘yes’ indicated a low risk of bias, whereas ‘no’ indicated a high risk of bias. Labeling an item as ‘unclear’ indicated an unknown or unclear risk of bias. Disagreements were resolved by discussion with a third author.

### Statistical analysis

Continuous data were pooled with a fixed-effects or random-effects model, according to the study heterogeneity. Data for BNP/NT-pro-BNP at the final follow-up evaluation were analyzed. Heterogeneity was quantitatively assessed with the *I*^2^ index. Effect sizes are reported as weighted mean differences (WMDs) and confidence intervals (CIs). Sensitivity analysis was conducted with the leave-one-out method to evaluate the influence of each study on the overall effect size. Potential publication bias was explored using Begg’s test and Egger’s test if there were at least five studies in the meta-analysis. Subgroup analysis was performed according to treatment duration, BMI and HbA1c at baseline. Statistical calculations for the meta-analysis were performed using RevMan 5.3 and STATA version 13.0.

## Results

### Flow of included studies

**Fig. 1:**
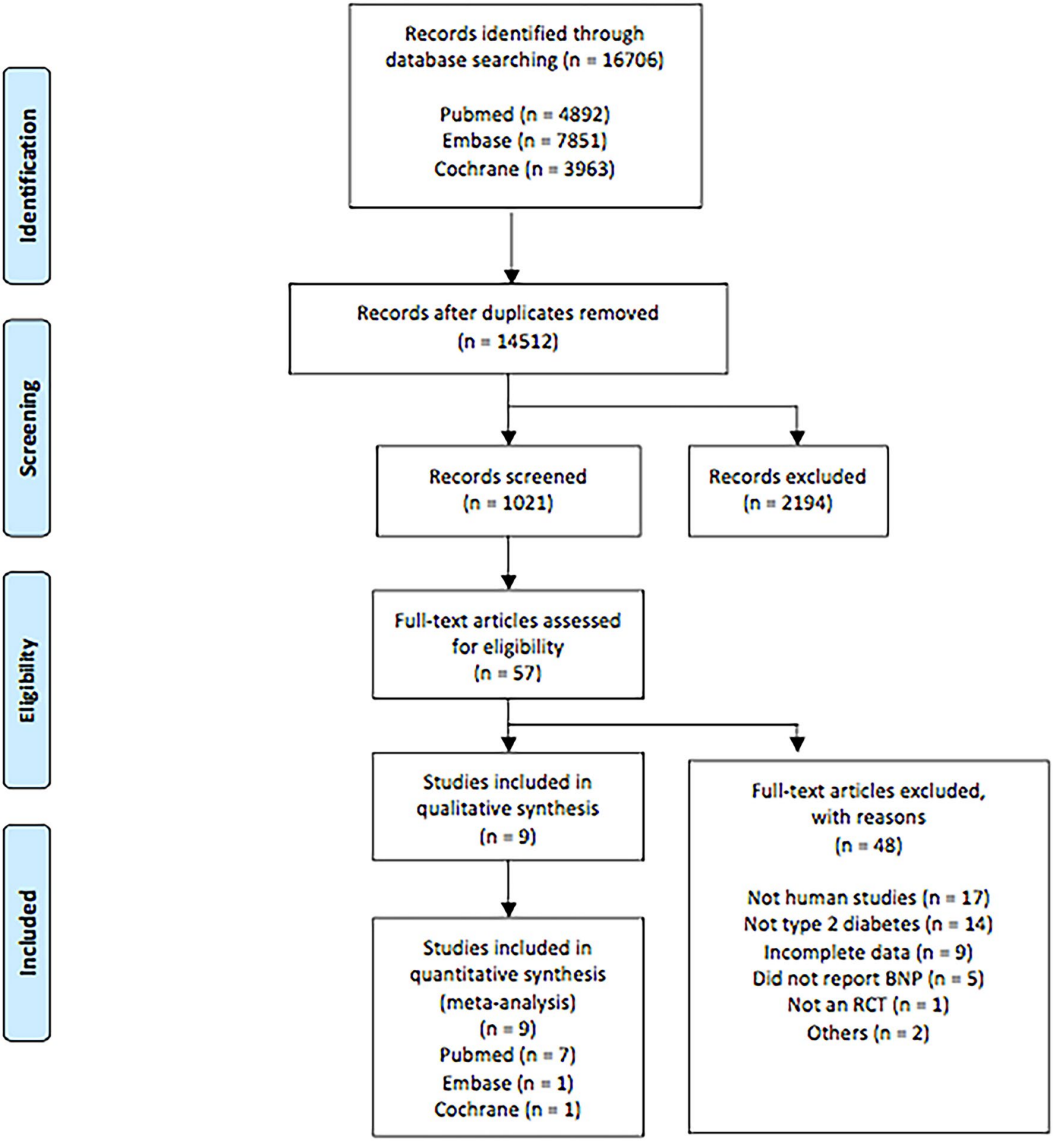
Flow chart of the number of studies identified and included into the meta-analysis

We identified 8690 published articles after systematic searching. After removal of inadequate studies, a total of 9 studies providing data for 6056 patients met the review eligibility criteria [10–18]. Of these patients, 3037 were treated with a DPP-4 inhibitor (222 with sitagliptin, 2701 with alogliptin, 68 with vildagliptin and 46 with linagliptin) in monotherapy or in addition to metformin or another antidiabetic agent, and 3019 were treated with placebo or control therapy. The flowchart of inclusion and exclusion is shown in Fig. 1.

### Characteristics of included studies

The characteristics of the included studies are presented in Table 1. These studies were published between 2015 and 2019. BNP and NT-pro-BNP were monitored in one study conducted by Fadini et al. [13]. Two studies evaluated the changes in BNP [11] and NT-pro-BNP [12], respectively, between patients treated with sitagliptin and those treated with active drugs, using three treatment arms. Out of the 9 studies, 4 set placebo as the control. Follow-up time ranged from 0.5 to 48 months. In the largest study, 5380 subjects were included, while the smallest study enrolled 20 subjects. Most of the studies compared sitagliptin with placebo or traditional antidiabetic agents. Active drugs or traditional antidiabetic agents discussed in this study included metformin, liraglutide, glargine, voglibose, glimepiride and dapagliflozin.

**Table 1:** Demographic characteristics of the studies included

Study, year	Location	Treatment arm (n)	HbA1c (%)	Male (n)	Age (years)	BMI (kg/m^2^)	Diabetes duration (years)	Treatment duration (months)	BNP/NT-pro-BNP (pg/mL)
Oe, 2015 [10]	Multicentre	sita:40 vog:40	7.1 ± 0.7 6.9 ± 0.5	30 21	67.8 ± 10.5 66.7 ± 9.8	27.7 ± 4.1 25.7 ± 4.3	4.0 ± 1.8 3.2 ± 1.5	6	39.0 ± 38.0 34.0 ± 35.0
Hiramatsu, 2018 [11]	Japan	sita:34 lina:32 lira:32	6.7 ± 0.7 6.7 ± 0.7 6.7 ± 0.6	NS	69.9 ± 8.5 69.0 ± 7.7 70.5 ± 5.7	24.2 ± 3.8 23.8 ± 4.8 23.5 ± 3.5	8.3 ± 8.3 8.3 ± 0.4 9.2 ± 7.0	48	83.9 ± 59.2 90.6 ± 68.6 91.6 ± 69.9
Arturi, 2016 [12]	Italy	sita:10 lira:10 glar:12	8.3 ± 0.9 8.2 ± 1.0 7.9 ± 0.8	6/10 7/10 9/12	60.5 ± 10 59.5 ± 9 60 ± 8	30.9 ± 2.8 33.2 ± 2.0 30.8 ± 6.0	NS	13	293.0 ± 46.0 310.0 ± 62.0 296.0 ± 60.0
Fadini, 2017 [13]	Italy	lina:22 pla:24	7.6 ± 0.2	33/46	63.7 ± 1.3	31.1 ± 0.7	NS	0.5	101.0 ± 93.2
Kitao, 2017 [14]	Multicentre	vild:48 met:48	7.3 ± 0.6 7.6 ± 1.1	26/48 31/48	62.0 ± 14.3 60.0 ± 18.0	25.7 ± 4.1 26.1 ± 4.7	5.4 ± 3.5 6.5 ± 3.5	3	31.0 ± 96.0 26.0 ± 68.0
Nomoto, 2016 [15]	Multicentre	sita:48 glim:55	7.4 ± 0.6 7.4 ± 0.4	33 29	62.0 ± 15.0 60.0 ± 8.0	25.7 ± 3.9 25.2 ± 3.5	NS	6.5	NS
Phrommintikul, 2019 [16]	Thailand	vild:24 dapa:25	8.2 ± 1.2 8.1 ± 1.4	12 11	63.9 ± 7.6 62.6 ± 8.3	24.9 ± 3.2 25.6 ± 3.0	> 5	6	955.8 ± 1853.7 399.9 ± 493.1
Yamada, 2017 [17]	Multicentre	sita:55 con:60	7.0 ± 0.6 6.9 ± 0.5	38/55 39/60	69.0 ± 8.0 69.0 ± 9.0	25.9 ± 3.3 24.8 ± 3.9	NS	24	111.5 ± 66.2 99.6 ± 60.9
Zannad, 2015 [18]	Multicentre	alog:2701 pla:2679	8.12 ± 1.12 8.15 ± 1.12	1637/2701 1631/2679	63.0 ± 4.7 62.0 ± 5.0	29.7 ± 2.5 29.5 ± 2.6	7.9 ± 13.0 6.8 ± 16.1	5	699.0 ± 500.7 630.0 ± 472.7

Values are expressed as mean  ±  SD.

*n* number of participants per group; *HbA1c* glycated haemoglobin; *sita* sitagliptin; *alog* alogliptin; *vild* vildagliptin; *lina* linagliptin; *met* metformin; *pla* placebo; *con* conventional treatment; *lira* liraglutide; *glar* glargine; *vog* voglibose; *glim* glimepiride; *dapa* dapagliflozin; *NS* not stated

### Quality evaluation

**Fig. 2:**
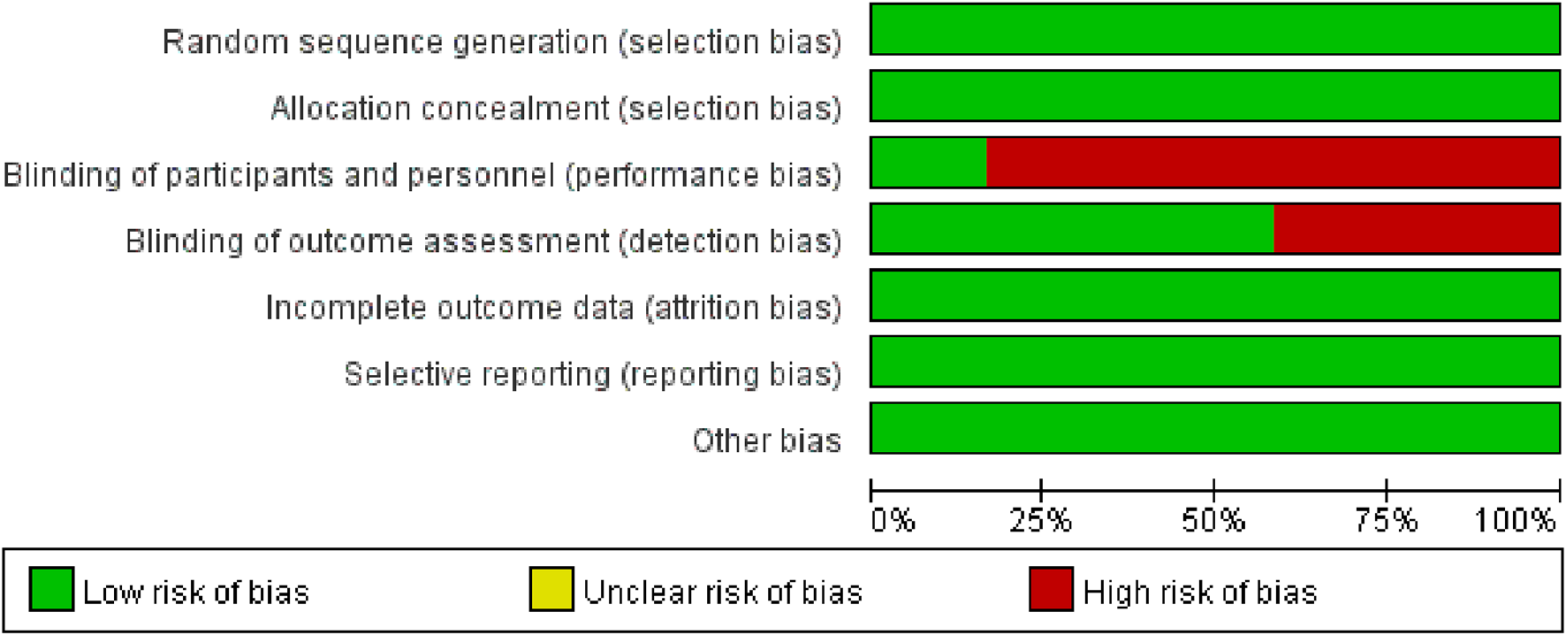
Risk of bias assessment in the studies identified for meta-analysis

Studies qualities were evaluated according to the scheme indicated by the Cochrane criteria. There was no unclear risk of bias in the studied items. All the studies had a randomized designed. Details on the items and their bias criteria across the studies are displayed in Fig. 2. Seven studies had performance bias due to the absence of blinding methods, whereas three studies had detection bias based on blinding of outcome assessment.

### Meta-analysis of the effect of DPP-4i treatment

**Fig. 3:**
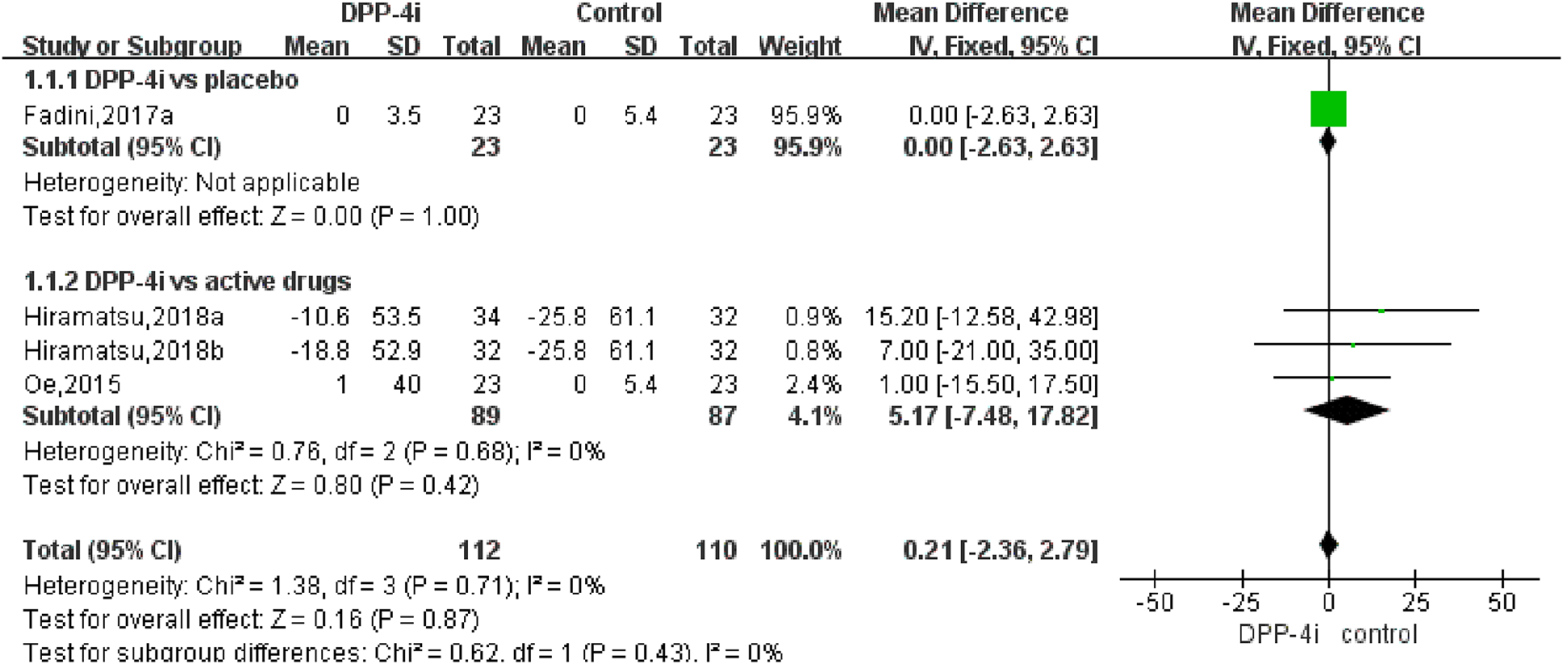
Forest plot for the impact of DPP-4i treatment versus placebo and active comparators on BNP concentrations

**Fig. 4:**
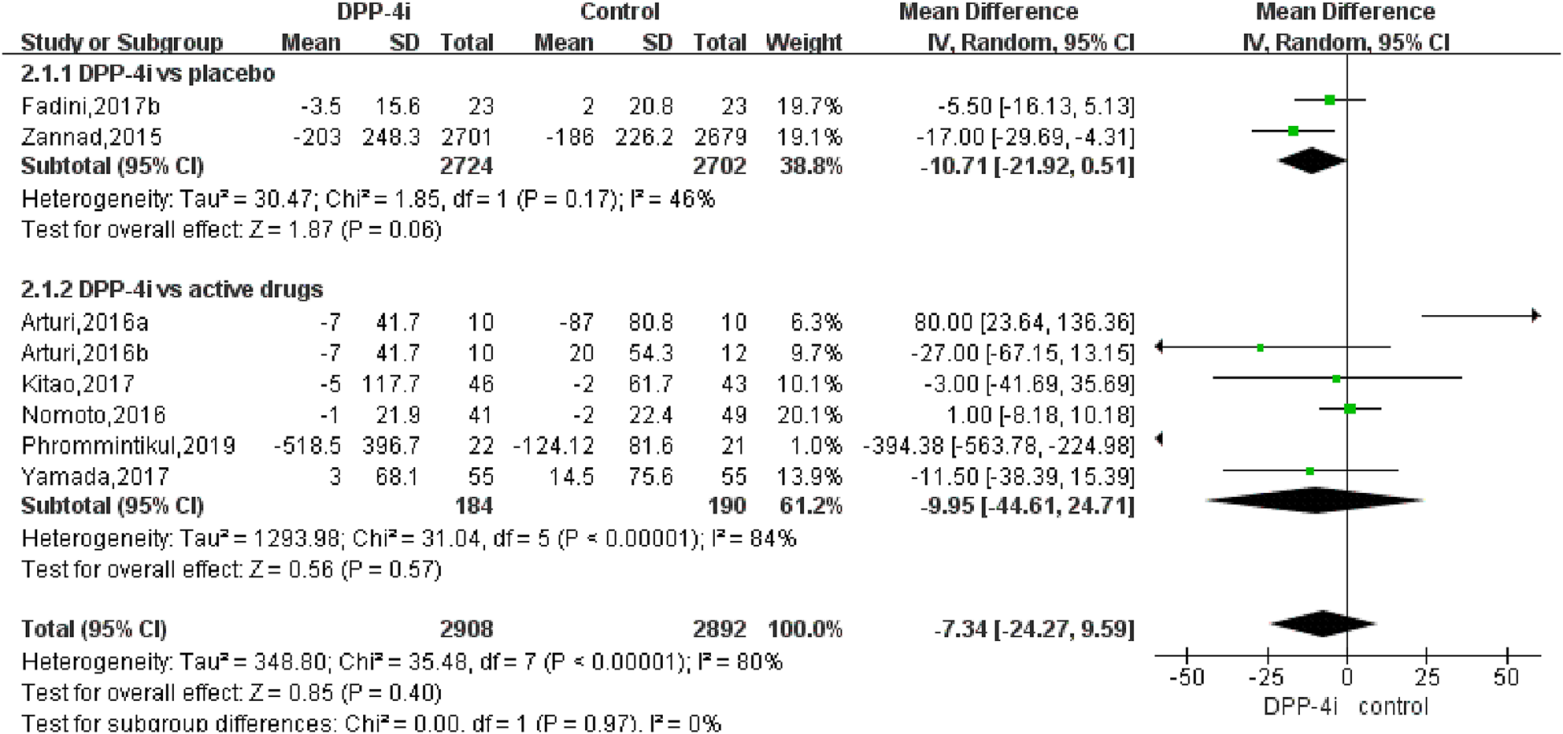
Forest plot for the impact of DPP-4i treatment versus placebo and active comparators on NT-pro BNP concentrations

**Fig. 5:**
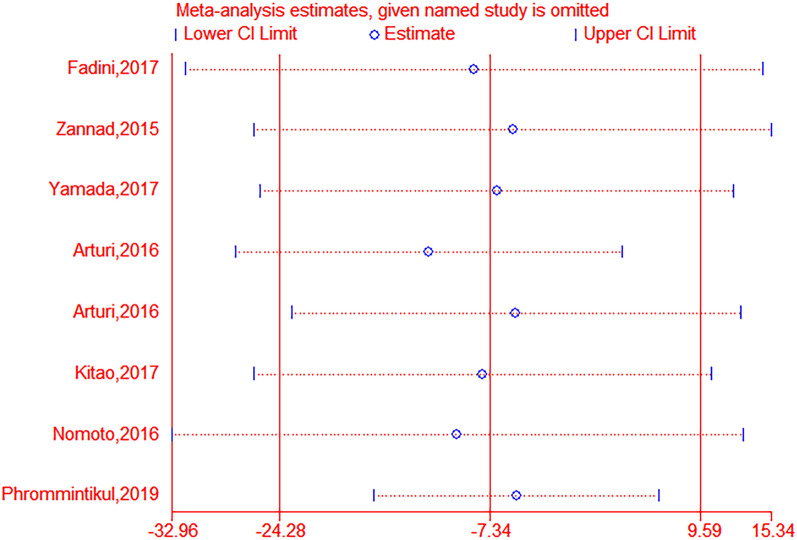
Leave-one-out sensitivity analysis for the impact of DPP-4i on plasma concentrations of NT-pro BNP

**Fig. 6:**
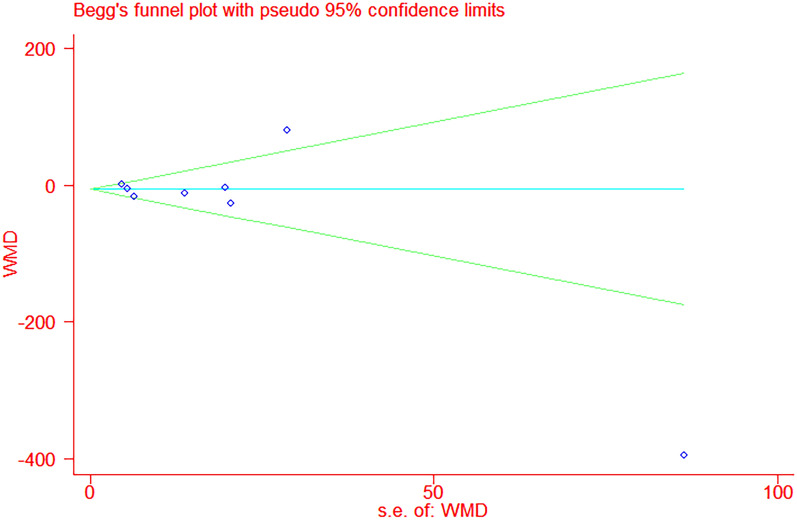
Assessment of publication bias in the meta-analysis of studies reporting the impact of DPP-4i on plasma concentrations of NT-pro BNP

The pooled analysis of the effect of DPP-4is on BNP levels yielded a difference of 0.21 pg/mL (95% CI − 2.36–2.79, *P*  = 0.71, *I*^2^  = 0%) and 5.17 pg/mL (95% CI − 7.48–17.82, *P*  = 0.42, I^2^  = 0%) compared to traditional antidiabetic agents (Fig. 3). The pooled analysis of the effect of DPP-4is on NT-pro-BNP levels yielded a difference of − 7.34 pg/mL (95% CI − 24.27 to 9.59, *P* = 0.40, *I*^*2*^ = 80%) and − 9.95 pg/mL (95% CI − 44.61–24.71, *P*  = 0.57, I^2^  = 84%) compared to traditional antidiabetic agents (Fig. 4). The pooled estimate of the modulating effect of DPP-4is on NT-pro-BNP was credible in the leave-1-out sensitivity analysis (WMD − 7.34 pg/mL, 95% CI − 24.27, 9.59, N  =  9 studies, heterogeneity *P*  = 0.395; Fig. 5). This confirmed that the significant difference across the studies was an overall effect of all the identified studies. In the subgroup analysis, baseline HbA1c, length of follow-up and BMI did not influence the effect of DPP-4is on NT-pro-BNP (Additional file 1: Figures S1–3). No publication bias was suggested by Begg’s test (*P*  = 0.62) or Egger’s test (*P*  = 0.86) across the 9 studies (Fig. 6).

## Discussion

The current study investigated the hypothesis that DPP-4 inhibition does not present an impact on BNP and NT-pro-BNP in T2DM. One important goal of antidiabetic agents in type 2 diabetes is to reduce cardiovascular complications and morbidity, in addition to lowering blood glucose levels. We found that DPP-4is did not significantly modulate BNP or NT-pro-BNP levels in T2DM compared to placebo or active antidiabetic agents.

BNP belongs to the natriuretic peptide family, which also includes atrial natriuretic peptide (ANP) and C-type natriuretic peptide (CNP). BNP binds to its receptor of natriuretic peptide type A receptor and exhibits a variety of actions, including vascular vasodilation and inhibition of renin secretion [19]. BNP is quickly metabolized to BNP 3–32 by DPP-4 in the plasma, which can be prevented by DPP-4is [20]. Under physiological conditions, plasma levels of BNP are low but increase in the pathophysiological state of heart failure. Administration of recombinant BNP effectively reduces afterload and exhibits a favorable effect on promoting natriuresis [21]. BNP can inhibit collagen synthesis and promote matrix metalloproteinase (MMP) activity through cGMP-PKG signaling, suggesting a role in regulating myocardial structure by modulating cardiac fibroblast function [22]. Plasma BNP activity seems to be absent in congestive heart failure [23]. It has been reported that BNP activity is modulated by DPP-4 in diabetic subjects with heart failure. DPP-4 activity is relatively high in patients with heart failure and thus modulates the pathophysiology of heart failure by acting on its substrate [24]. In fact, DPP-4 rapidly cleaves BNP 1–32 to produce BNP 3–32, and the latter shows reduced activity in modulating urinary sodium excretion and urine flow. However, the exact biological activity of BNP 3–32 remains uncertain. The two major subtypes of BNP in the plasma are BNP 1–32 and BNP 3–32, while we evaluated the change of total BNP levels before and after intervention in this study. Further studies will be needed to accurately determine the modulating effect of DPP-4is on different subtypes of BNP. DPP4-deficient rat models showed better cardiac performance, with a decrease in infarct size and cardiac injury biomarkers of BNP [25]. In another study, DPP-4 inhibition with vildagliptin or sitagliptin did not increase or decrease BNP levels in patients with T2DM [37].

BNP concentrations are reduced in people with obesity, insulin resistance, and diabetes, and this deficiency may contribute to their cardiovascular risk [13]. The N-terminal pro-brain natriuretic peptide (NT-pro-BNP) is biologically inert and is secreted in the plasma, like BNP. BNP and NT-pro-BNP are used in the diagnosis of congestive heart failure in elderly individuals and seem to have comparable diagnostic value [26]. Elevation of NT-pro-BNP levels shows a strong correlation with a poor prognosis of elderly chronic heart failure patients [27]. Plasma levels of NT-pro-BNP also increase in obese and diabetic patients, which might be related to chronic low-grade inflammation and insulin resistance [28]. NT-pro-BNP concentrations are affected by the presence of comorbidities such as T2DM and acute coronary syndrome. Serial monitoring of NT-pro-BNP might be useful for identifying patients with a higher risk of heart failure in patients with T2DM and ischemic heart diseases [29]. NT-pro-BNP is a better short-term independent predictor of cardiovascular mortality than C-reactive protein and albumin excretion rate in elderly patients with T2DM, according to the Casale Monferrato population-based study [30].

Under pathological conditions, the mRNA encoding BNP can rapidly synthesize BNP precursor (pre-pro-BNP) and be split into NT-pro-BNP [31]. Although a number of studies have been carried out to investigate the effect of DPP-4is on modulating BNP and NT-pro-BNP in diabetes, no firm conclusions have been drawn. Compared to conventional antidiabetic regimens, add-on sitagliptin treatment showed no significant difference in changing plasma levels of NT-pro-BNP according to the subanalysis of the PROLOGUE study [17]. In another study, DPP-4 inhibition with vildagliptin or sitagliptin did not increase or decrease BNP levels in T2DM patients [37]. In a multicenter, randomized, double-blind trial, alogliptin presented no significant effect on the composite event of cardiovascular death and hospital administration among heart failure in patients with T2DM. However, plasma levels of NT-pro-BNP decreased significantly after treatment with alogliptin [18]. Our results are consistent with a previous study, in which sitagliptin did not regulate vascular function by affecting the degradation of GLP-1 and BNP in healthy participants [32]. Moreover, in a preclinical study, DPP-4is seemed to downregulate the level of neprilysin, thus reducing the degradation of BNP and ANP [33]. However, in another randomized cross-over trial in patients with T2DM, acute treatment with linagliptin led to no significant change in BNP or NT-pro-BNP level compared to placebo in patients with or without chronic kidney disease. This suggested that changes in NT-pro-BNP over the long run most likely reflect the natural history of heart failure rather than an effect of antidiabetic treatment [13].

DPP4i-treated T2DM patients had lower risks for cardiovascular events, including heart failure, myocardial infarction and ischemic stroke, than non-DPP4i users [34]. In the SAVOR-TIMI 53 study, saxagliptin was found to be correlated with an increased risk of hospitalization for heart failure [35]. However, no increased risk of hospitalization for heart failure was found in patients treated with DPP-4is through an analysis of Taiwan’s National Health Insurance claims database, which included a total of 239,669 T2DM patients treated with sitagliptin, saxagliptin or vildagliptin from 2011 to 2013. Three DPP-4is have been proven to be safe in terms of the risk of heart failure at present. Saxagliptin had a similar risk (HR: 0.98, 95% CI 0.91–1.06) as sitagliptin, while vildagliptin was associated with a lower risk of heart failure (HR: 0.85, 95% CI 078–0.93) [36]. In another observational study including 962 hospitalized heart failure patients, DPP-4is seemed to improve cardiac and all-cause mortality in hospitalized heart failure patients with diabetes mellitus [37]. According to a pooled meta-analysis including 90 randomized clinical trials, DPP-4is showed a similar safety profile as placebo in patients with type 2 diabetes. There was only weak evidence for an increased risk of heart failure [38].

The current study is the first pooled analysis demonstrating the effect of DPP-4is on serum BNP and NT-pro-BNP concentrations in patients with T2DM. We performed a sensitivity analysis on the included trials and surprisingly found that DPP-4is caused no significant increase in the plasma level of BNP or NT-pro-BNP.

Our study might have some limitations. First, the methods of measuring BNP or NT-pro-BNP might be different in each study, which could cause an inevitable bias. Second, the studies had differences in the baseline BNP or NT-pro-BNP values in patients with T2DM and in whether they had received cardiovascular therapy, which also could have had an effect on the pooled analysis. Third, few trials were included in the pooled analysis, which could cause a publication bias. Further studies with more trials should be performed to verify any associations between DPP-4is and BNP/NT-pro-BNP. Fourth, we only investigated the impact of DPP-4is on BNP/NT-pro-BNP concentrations, while the effect of DPP-4is on the other biomarkers of heart failure should be explored with further studies. Last, the impact of DPP-4is on BNP and NT-pro-BNP in the long run should also be taken into consideration, which should be studied in trials with long-term DPP-4i treatment.

## Conclusions

In conclusion, DPP-4is have no significant effects on the BNP or NT-pro-BNP level in patients with T2DM. These agents are relatively well tolerated when used as monotherapy or in combination with traditional antidiabetic agents in diabetes. Data on long-term effects are needed in patients with T2DM with risks of cardiovascular disease, such as heart failure. In addition, BNP and NT-pro-BNP monitoring should be done in T2DM patients with cardiovascular disease who take antidiabetic agents such as DPP-4is.

## Supplementary Information

**Additional file 1: Figure S1.** Forest plot for the impact of DPP-4i treatment versus comparators on serum concentrations of NT-pro BNP in subgroups of trials with HbA1c levels of  < 8.0% and  ≥ 8.0%.

**Additional file 2: Figure S2.** Forest plot for the impact of DPP-4i treatment versus comparators on serum concentrations of NT-pro BNP in subgroups of trials with treatment durations of  < 6 months and  ≥ 6 months.

**Additional file 3: Figure S3.** Forest plot for the impact of DPP-4i treatment versus comparators on serum concentrations of NT-pro BNP in subgroups of trials with ages of  < 60 years and   60 years.

## Data Availability

All data generated or analyzed during this study are included in this published article (and its Additional files).

## Abbreviations

DPP-4is: Dipeptidyl peptidase-4 inhibitors
T2DM: Type 2 diabetes mellitus
RCTs: Randomized controlled trials
BNP: Brain natriuretic peptide
NT-pro BNP: N-terminal probrain natriuretic peptide
CVD: Cardiovascular disease
CI: Confidence intervals
SD: Standard deviations
WMD: Weighted mean difference
GLP-1: Glucagon-like peptide 1
GIP: Glucose-dependent insulinotropic polypeptide
ANP: Atrial natriuretic peptide
CNP: C-type natriuretic peptide (CNP)
MMP: Matrix metalloproteinase
BNP: Brain natriuretic peptide
NT-pro BNP: N-terminal probrain natriuretic peptide
