# Supplementary figures and images for: Impact of DPP-4 inhibitors on plasma levels of BNP and NT-pro-BNP in type 2 diabetes mellitus
Source: Diabetol Metab Syndr. 2022 Feb 14;14:30. doi: 10.1186/s13098-022-00797-x (PMC8842815; doi:10.1186/s13098-022-00797-x)

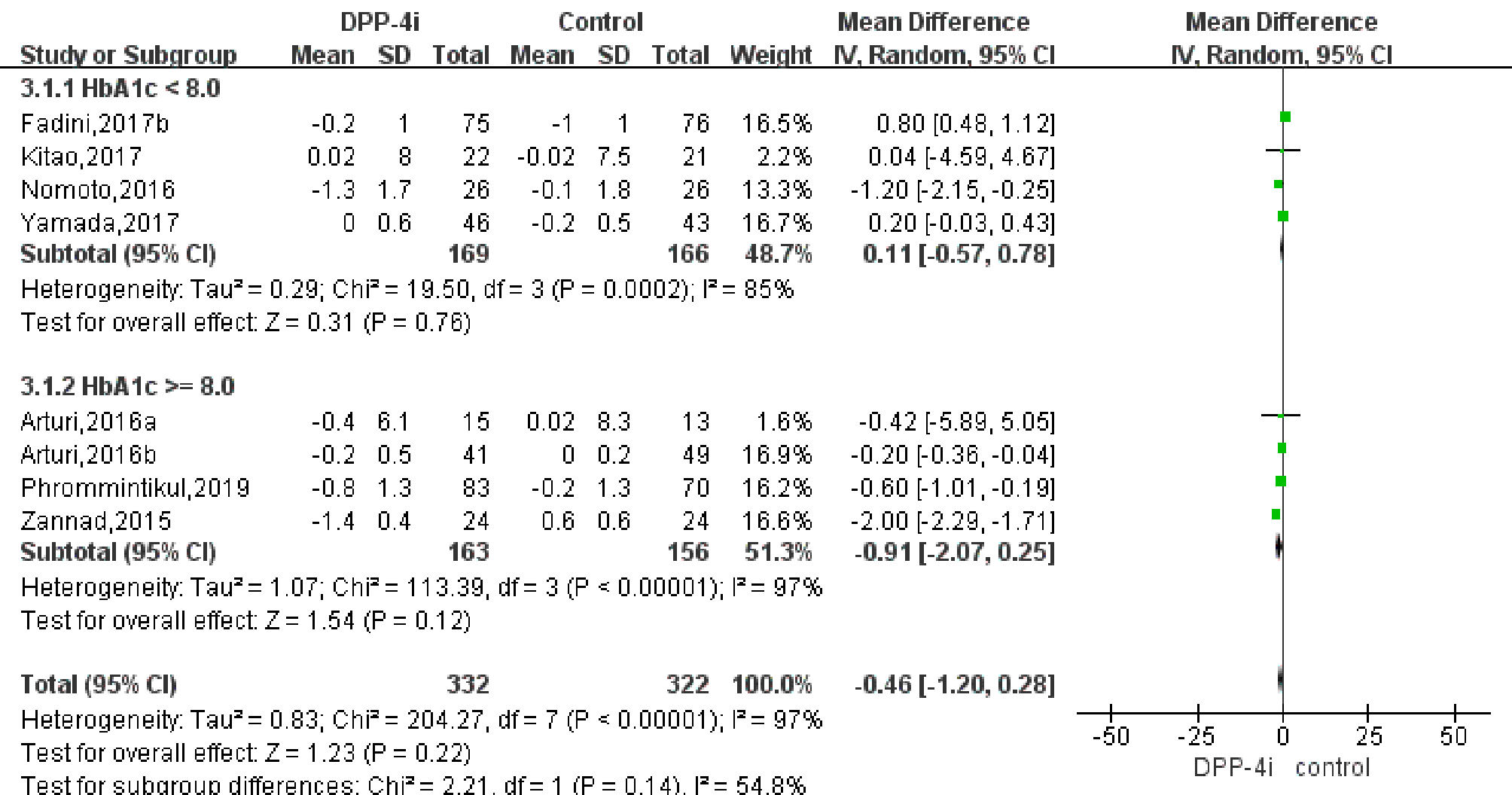

Supplement: Supplementary file 1 — Additional file 1: Figure S1. Forest plot for the impact of DPP-4i treatment versus comparators on serum concentrations of NT-pro BNP in subgroups of trials with HbA1c levels of < 8.0% and ≥ 8.0%. [file 13098_2022_797_MOESM1_ESM.png]

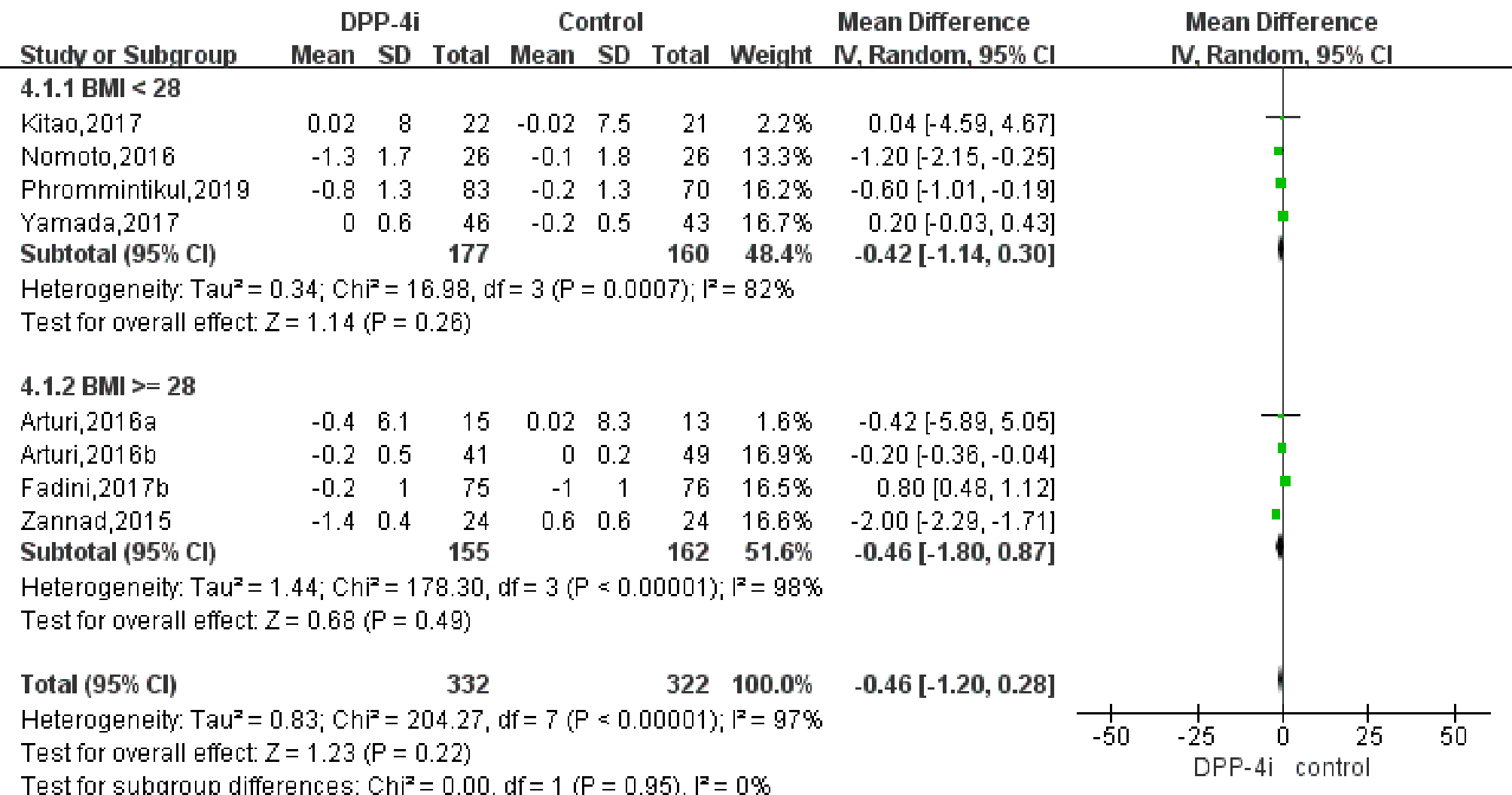

Supplement: Supplementary file 2 — Additional file 2: Figure S2. Forest plot for the impact of DPP-4i treatment versus comparators on serum concentrations of NT-pro BNP in subgroups of trials with treatment durations of < 6 months and ≥ 6 months. [file 13098_2022_797_MOESM2_ESM.png]

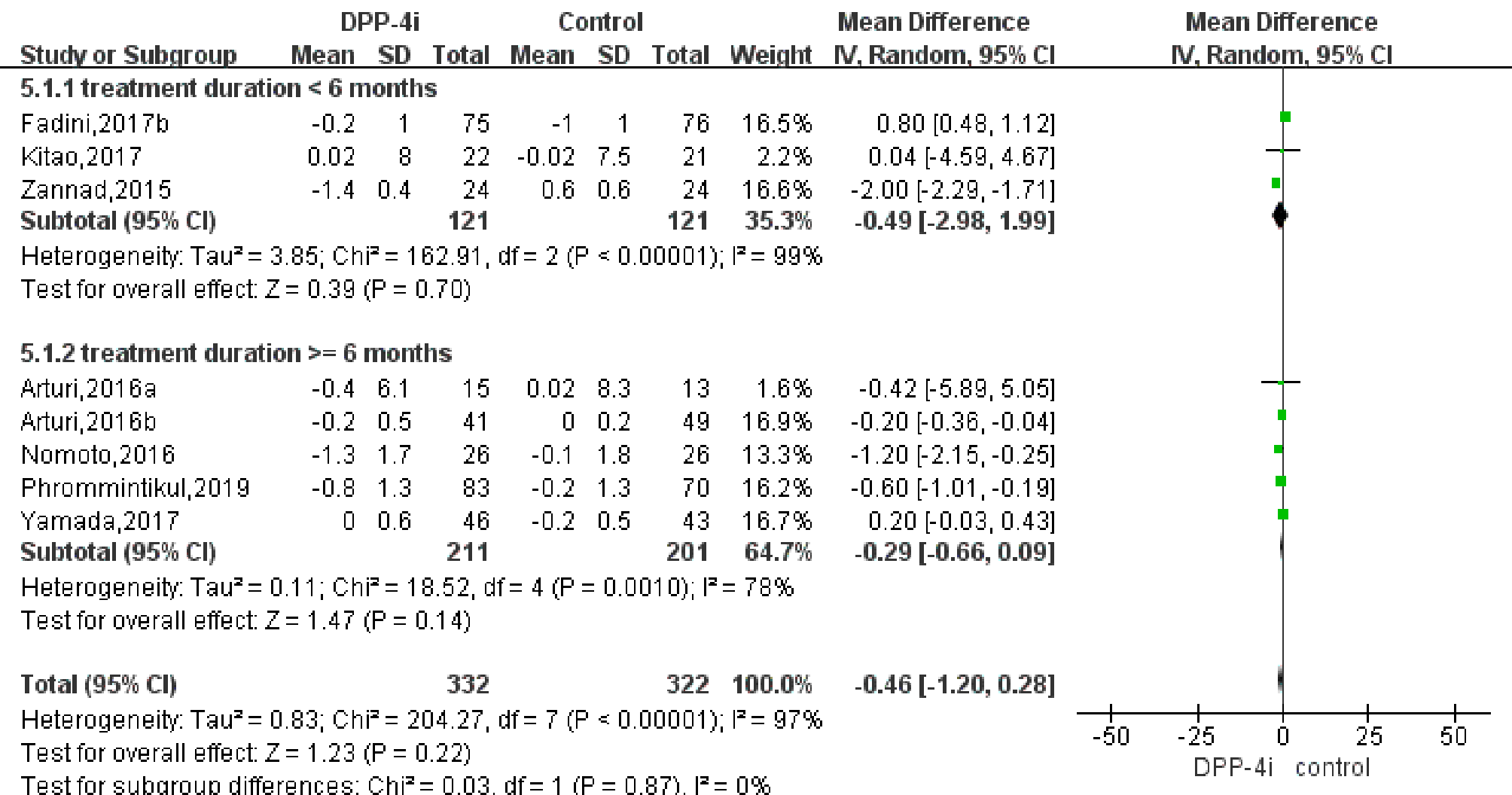

Supplement: Supplementary file 3 — Additional file 3: Figure S3. Forest plot for the impact of DPP-4i treatment versus comparators on serum concentrations of NT-pro BNP in subgroups of trials with ages of < 60 years and 60 years. [file 13098_2022_797_MOESM3_ESM.png]
